# New Multidrug Efflux Systems in a Microcystin-Degrading Bacterium *Blastomonas fulva* and Its Genomic Feature

**DOI:** 10.3390/ijms231810856

**Published:** 2022-09-17

**Authors:** Long Jin, Chengda Cui, Chengxiao Zhang, So-Ra Ko, Taihua Li, Feng-Jie Jin, Chi-Yong Ahn, Hee-Mock Oh, Hyung-Gwan Lee

**Affiliations:** 1 College of Biology and the Environment, Nanjing Forestry University, Nanjing 210037, China; 2 Cell Factory Research Centre, Korea Research Institute of Bioscience & Biotechnology (KRIBB), Daejeon 34141, Korea

**Keywords:** *Blastomonas fulva*, microcystin degradation, multidrug efflux system, RND pumps, MFS, ABC transporter

## Abstract

A microcystin-degrading bacterial strain, *Blastomonas fulva* T2, was isolated from the culture of a microalgae *Microcystis*. The strain *B. fulva* T2 is Gram-stain-negative, non-motile, aerobic, non-spore-forming and phototrophic. The cells of *B. fulva* T2 are able to grow in ranges of temperature from 15 to 37 °C, with a pH of 6 to 8 and a salinity of 0 to 1% NaCl. Here, we sequenced the complete genome of *B. fulva* T2, aiming to better understand the evolutionary biology and the function of the genus *Blastomonas* at the molecular level. The complete genome of *B*. *fulva* T2 contained a circular chromosome (3,977,381 bp) with 64.3% GC content and a sizable plasmid (145.829 bp) with 60.7% GC content which comprises about 3.5% of the total genetic content. A total of 3842 coding genes, including 46 tRNAs and 6 rRNAs, were predicted in the genome. The genome contains genes for glycolysis, citric acid cycle, Entner–Doudoroff pathways, photoreaction center and bacteriochlorophyll*a* synthesis. A 7.9 K gene cluster containing *mlrA*, *mlrB*, *mlrC* and *mlrD*_1,2,3,4_ of microcystin-degrading enzymes was identified. Notably, eight different efflux pumps categorized into RND, ABC and MFS types have been identified in the genome of strain T2. Our findings should provide new insights of the alternative reaction pathway as well as the enzymes which mediated the degradation of microcystin by bacteria, as well as the evolution, architectures, chemical mechanisms and physiological roles of the new bacterial multidrug efflux system.

## 1. Introduction

**Figure 1:**
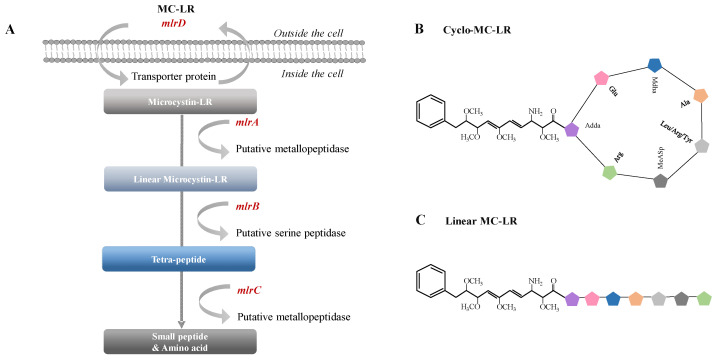
(**A**) The degradative pathway of MC-LR and the formation of intermediate products. (**B**) A simplified cyclo-structure of MC-LR and (**C**) linear structure of MC-LR. The colored pentagram symbols indicate amino acids that form the structure of MCs. Adda,3-amino-9-methoxy-2,6,8-trimethyl-10-phenyl-deca-4,6-dienoic acid.

Hepatotoxic microcystins (MCs) are very stable against heat, pH, proteases and other hepatotoxic substances produced by *Microcystis*, which is the most well-known freshwater *Cyanobacteria* that causes harmful algal blooms [1,2,3,4]. MCs are cyclic heptapeptides structurally containing seven amino acids. MC variants contain different amino acid residues at two positions, which make the differentiation between variants of MCs. In MC-LR, two variable elements are leucine and arginine. MC-LR (microcystin–leucine–arginine) is most toxic among the MCs produced by *Cyanobacteria*. Due to their ring structure, MCs are highly resistant to degradation; however, they can be degraded by specific enzymes [2,5,6,7,8]. The *mlrA* gene encodes a hydrolytic enzyme that opens the cyclic peptide of MC; the *mlrB* gene encodes metallopeptidase that degrades linearized MCs; the *mlrC* gene encodes an enzyme-like serine peptidase that breaks linearized MCs or oligopeptides; and the *mlrD* gene encodes a putative transporter protein involved in the active transport of MCs (Figure 1).

Investigations tend to focus on microbial efflux systems that have direct clinical relevance for humans and animals. Efflux systems that are not clinically important can be uncovered with relative ease using Omics-technologies, but their actual activities and significance in bacterial metabolism remain poorly understood and underexplored. As a result, research into new bacterial efflux pump systems and the proteins that cooperate with them is critical for understanding their evolution, architectures, molecular mechanisms and precise physiological roles.

Antimicrobial resistance is a global issue and raises questions regarding how to handle antimicrobials. Overusing antibiotics as growth promoters or preventives in humans and animals is one of the major causes of antibiotic resistance. Heavy metals and chemical fertilizers may drive environmental strains to express multidrug efflux pumps, resulting in cross-resistance [9,10,11,12]. In human and veterinary pathogenesis, bacterial multidrug efflux pumps defend microorganisms from antimicrobials. The physiological functions of bacterial multidrug efflux pumps can be described as follows: transport of antimicrobial peptides; protection against mammalian bile acids/salts and hormones; protection against plant-derived toxins; tolerance toward pH, salt, heavy metals and aromatic hydrocarbons; protection against oxidative and nitrosative stress; cell-to-cell signaling; bacterial biofilm formation, etc. [13,14,15,16,17,18,19,20]. Many, if not all, of these pumps perform physiological purposes other than protecting bacteria from antimicrobials [21,22,23,24]. Apart from antibiotics, efflux pumps can extrude a wide variety of non-antibiotic substrates, including heavy metals, organic compounds, plant-produced chemicals, quorum sensing signals and bacterial metabolites [25,26,27].

*Blastomonas* can grow aerobically, anaerobically or phototrophically [28,29,30,31,32,33]. The genome of this genus bears the *puf* genes, which code for proteins of the L and M subunits of the reaction center complex and LH1 complex [28,29,32,34]. *Blastomonas* species are Gram-negative, aerobic, non-spore-forming, reproduce via budding or asymmetric cell division, generate carotenoids and bacteriochlorophyll*a* and contain ubiquinone-10 as the primary respiratory quinone [28,29,30,31,32]. Thus far, two *Blastomonas* genomes have been analyzed and published [35,36]. In order to better understand its metabolism, we present the complete genome sequence of *B. fulva* T2, a microcystin-degrading strain with several multidrug efflux pumps isolated from a *Microcystis* culture. Our results focus on genes encoding proteins of major pathways of carbon metabolism, as well as genes related to photosynthesis, microcystin degradation and multidrug resistance system.

## 2. Results and Discussion

### 2.1. General Genomic Features

**Figure 2:**
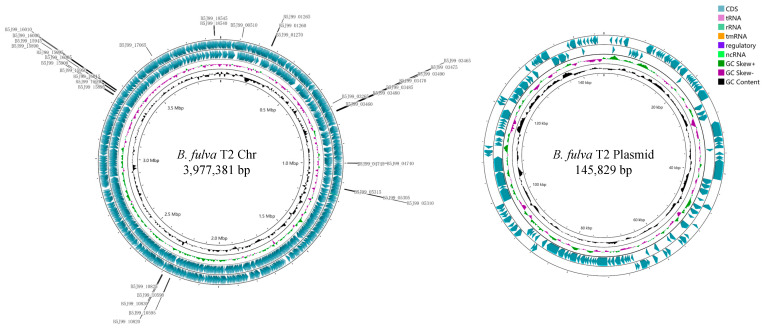
Graphic representation of circular genome plot of strain *B. fulva* T2. The locations of genes involved in multidrug efflux systems are indicated at the outside of the map. The circles are organized from outside to inside, with the first and second circles representing protein-coding regions (CDS). The third and fourth circles represent GC skew and G+C variation, respectively.

General features of strain *B. fulva* T2 were summarized in Table 1. The genome size of strain T2 was 4,123,210 bp with a DNA G+C value of 64.2 mol %, consisting of a single circular chromosome (CP020083) of 3,977,381 bp and a single circular plasmid (CP020084) of 145,829 bp (Figure 2). Of the 3887 genes identified in the total genome, 3762 were protein-encoding genes; 6 were ribosomal, 46 were transfer RNAs and 4 were noncoding RNAs.

### 2.2. Carbon Metabolism and Phototrophic Related Genes

**Figure 3:**
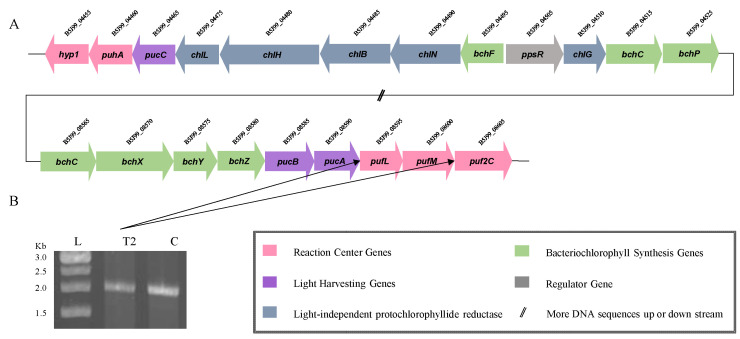
(**A**) Photosynthetic gene cluster of strain *B. fulva* T2. (**B**) PCR amplification products of *puf* gene containing the L and M subunits. Lanes: L, size marker; C, control strain *B. natatoria* DSM 3183^T^. Color key: pink, *puf* gene (photosynthetic reaction center subunits); *puhA* gene (photosynthetic reaction center H subunit): purple, *puc* gene (light-harvesting subunits); *hyp1* gene (putative photosynthetic complex assembly protein): blue-gray, *chl* gene (light-independent protochlorophyllide reductase): green, *bch* genes (bacteriochlorophyll synthesis).

Genes encoding enzymes of a complete glycolysis and the citric acid cycle were discovered in *B. fulva* T2, as well as genes for the pentose phosphate and Entner–Doudoroff pathways. Like other *Blastomonas* members, the key genes of the Calvin–Benson cycle RuBisCO (ribulose 1,5-bisphosphate carboxylase/oxygenase) are absent in the *B. fulva* T2. The anaplerotic CO_2_ assimilation via the activity of phosphoenolpyruvate carboxykinase (*pckA*) is an important mechanism of non-autotrophic CO_2_ fixation. Interestingly, the *pckA* (B5J99_17370) gene is present in the genome of strain T2. Members of the genus *Blastomonas* are characterized to produce bacteriochlorophyll*a* and contain light-harvesting complexes. The genomes of strain T2 contain key photosynthesis genes encoding the light-harvesting protein beta and alpha subunits (*pucBA*) as well as reaction center L, M, C and H subunits (*pufLM2C* and *puhA*). PCR amplification also detected the *puf* genes, which code for proteins of the L and M subunits of the active photosynthetic reaction center and of the core light-harvesting complex (Figure 3). Additionally, there are genes encoding for bacteriochlorophyll (*bchFCXYZ*) and light-independent protochlorophyllide reductase (*chlLBN*).

### 2.3. Microcystin Degradation and Related Genes

**Figure 4:**
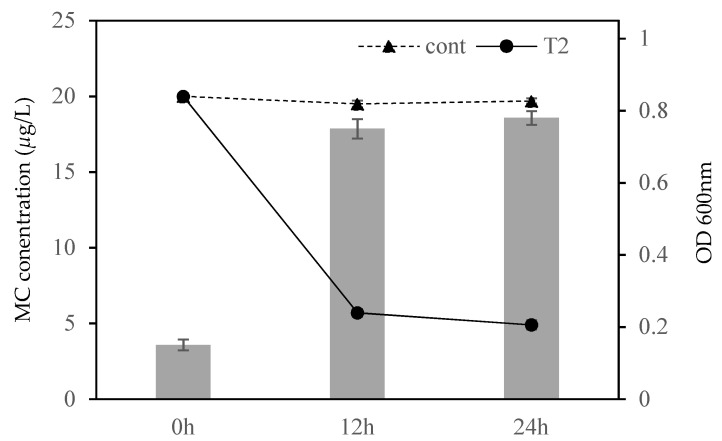
Degradation of MC-LR by bacterial cells of *B. fulva* T2. Filled circle symbols represent *B. fulva* T2 incubated on MC-LR, and triangle symbols represent control groups (medium without bacterial cells). Error bars represent standard deviations of three individual replicates.

The results demonstrate that both total and extracellular microcystin concentrations were much lower compared to the control group (Figure 4). The results indicate that the average degradation rate of MC-LR was 0.208 mg/L/d.

**Figure 5:**
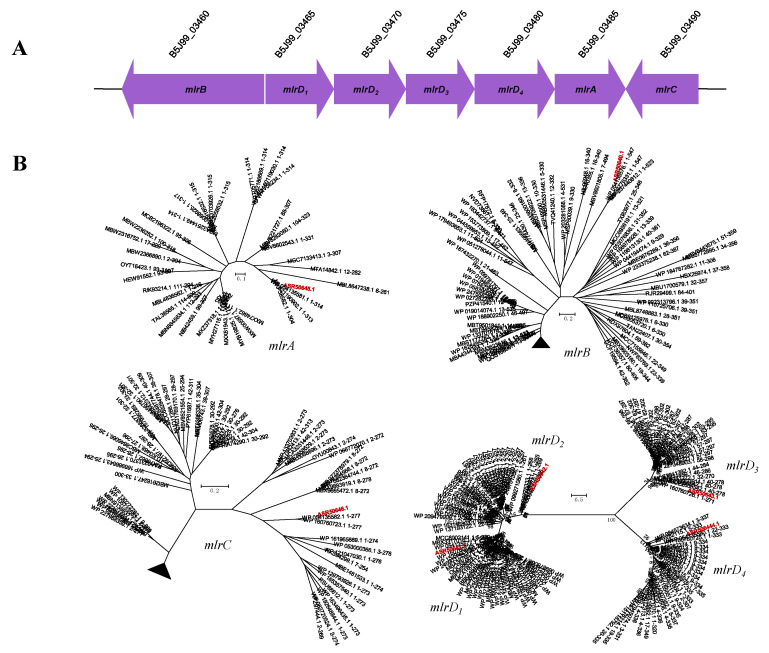
(**A**) Genetic organization of *mlrBDAC* and localization of genes involved in MC-LR degradation. (**B**) Phylogenic trees of *mlr*-like gene sequences of *B. fulva* T2 and other available *mlr* sequences. The minimum-evolution trees were constructed based on translated amino acid sequences. The scale bar indicates the number of amino acid sequence substitutions per site. Bootstrap values were calculated applying 1000 replicates. The GenBank accession numbers are shown in parentheses. The sequences derived from *B. fulva* T2 were indicated in red.

**Figure 6:**
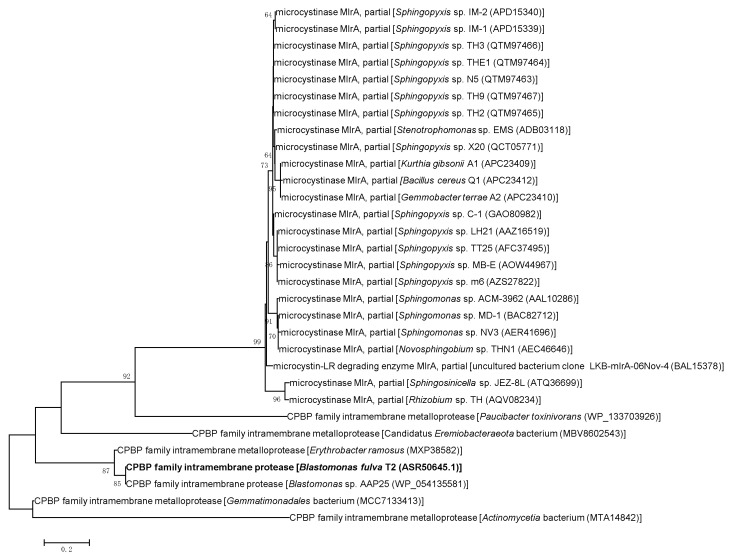
The minimum-evolution-based phylogenetic analysis of the translated amino acid sequences in the partial microcystinase MlrA and CPBP family metalloproteases. The bootstrap values were calculated based on 1000 replicates, and scale bars indicate 0.2 changes per position. The accession numbers of the corresponding sequences were given in parentheses.

The significant finding was that *B. fulva* T2 could completely degrade MC-LR (Figure 4). To obtain unknown functional genes encoding enzymes responsible for MC-LR degradation, the *mlrA*, *mlrB*, *mlrC* and *mlrD* homolog genes of *B. fulva* T2 were further analyzed through the genomic analysis. A genome-oriented study revealed that *B. fulva* T2 was found to harbor homologs of the gene cluster *mlrBD_1,2,3,4_AC* (B5J99_03460 to B5J99_03490, Figure 5A), which were responsible for the conversion of MC-LR to Adda [5]. The *mlrA* gene encodes a neutral metalloprotease with optimal function at pH 7.6 capable of the hydrolytic cleavage of the cyclic structure of microcystin, hence reducing its toxicity [37]. The sequences of the *mlrA* homolog gene (B5J99 03485) and 315 putatively translated amino acids have been determined (GenBank accession number ASR50645.1). The coding region of the *mlrA* gene had a G+C content of 65.9%. The nucleic acid and putative protein sequences analysis showed low similarities of the MlrA peptidase to mircocystin degrading enzymes (Table 2). The alignment of these enzymes exhibits 37.9–42.2% amino acid sequence and 51.8–95.4% nucleic acid sequence similarities. Therefore, a phylogenetic analysis for the translated amino acid sequence of the *mlrA* homolog along with the microcystinase MlrA was performed, which is the first key degradative enzyme responsible for cleaving the cyclic MC into the linearized MC in the pathway of MC degradation (Figure 5B). The phylogenetic tree showed that the *mlrA* gene formed a clade with the CPBP family (CAAX Proteases and Bacteriocin-Processing enzymes) intramembrane metalloproteases (Figure 6). The MlrA as membrane protein belongs to the CPBP family converting cyclic MC to linear, and the analysis revealed that the gene may encodes putative microcystinase MlrA which breaks the conventional hydrogen bond to reduce its toxicity.

The *mlrB* gene (B5J99_03460) is located downstream of the *mlrA* and *mlrD_1,2,3,4_* genes encoding proteins which cleave linear MC-LR to a tetrapeptide degradation product and possibly characterized as serine peptidase [5]. The *mlrB* gene sequence along with the putative translated amino acid sequence of 548 residues was determined (GenBank accession number ASR50640.1). The coding region had a G+C content of 68.2%. The pairwise analysis established low similarities of the MlrB peptidase to mirocystin-degrading enzymes (Table 2). Pairwise alignment of these enzymes exhibits 27.1–31.3% amino acid sequence and a 49.0–49.9% nucleic acid sequence similarities.

The *mlrC* gene (B5J99_03490) encoding a metallopeptidase MlrC is located next to the *mlrA* homolog and responsible for the hydrolysis of Adda-Glu in the degradation of tetrapeptide to Adda [8], and a similar gene organization was observed in genomes of *Sphingopyxis* sp. C-1 and *Sphingosinicella microcystinivorans* B9 (Figure 5A) [38,39]. Like *mlrB*, the *mlrC* gene is transcribed in the opposite direction to the *mlrA* and *mlrD* genes (Figure 5A). The *mlrC* gene (B5J99_03490) sequence along with the putative translated amino acid sequence of 278 residues was determined (GenBank accession number ASR50646.1), and the coding region had a G+C content of 65.1%. The predicted proteins exhibited no significant matches with previously characterized MlrC, but the enzyme was identified as aminopeptidase, which may functionally work the same way as *mlrC*.

Previously, Bourne et al. (2001) showed that the function of *mlrD* is unclear [5]. It was postulated that the *mlrD* gene was involved in the transfer of either parent MC into the cellular environment or MC degradation products out of the cell. Within the 7.9 kb gene cluster, four *mlrD* gene homologs (B5J99_03465-03480) were identified just downstream of *mlrA*, and this is not an usual gene structure previously observed. The sequences of nucleic acid and amino acid were applied at the NCBI database using the BLASTN and BLASTP program. As a result, high sequence similarity suggested that the proteins belong to the ABC-type dipeptide/oligopeptide/nickel transport system as permease components. This family of proteins is involved in the transport and metabolism of amino acids and inorganic ions. As with *mlrC*, there were no significant matches between the predicted proteins and previously identified *mlrD* genes in the database. However, the function of the putative proteins was identical to that of microcystinase MlrD, so it is possible that one of them acts as MlrD or that all four genes work together to complete their functions.

Strain T2, which has exhibited a significant capacity for microcystin degradation, possesses a cluster of *mlr* gene homologs. It is considered to encode different hydrolytic proteins potentially involved in the initial or intermediate steps of MC degradation. The enzymes MlrA, MlrB and MlrC possibly undertake aminolysis processes, whereas the MlrD proteins intake or uptake small peptides that are produced. To date, the whole genomic sequences of only five MC-LR-degrading bacterial strains, namely *Novosphingobium* sp. THN-1, *Novosphingobium* sp. MD-1, *Sphingopyxis* sp. C-1, *Sphingopyxis* sp. YF1 and *Sphingosinicella microcystinivorans* B9 have been obtained [38,39,40,41,42]. Moreover, there are rare reports about functional genes for MC degradation with the exception of the *mlrBDAC* gene cluster. Although Okano et al. reported that *Novosphingobium* MD-1 possesses *mlrE* and *mlrF* genes participating in MC degradation, further research is needed to assess the MC-degrading function and gene structures. Searching novel *mlr* gene homologs help scientists better understand their functional characteristics and metabolic pathways. Intriguingly, PCR studies failed to amplify genes encoding hydrolytic enzyme (*mlrA*), metallopeptidase (*mlrB*), suspected serine peptidase (*mlrC*) and putative transporter protein (*mlrD*), suggesting that unique enzymes or a pathway for MC-LR might exist in this strain.

### 2.4. Multidrug Efflux Systems

**Figure 7:**
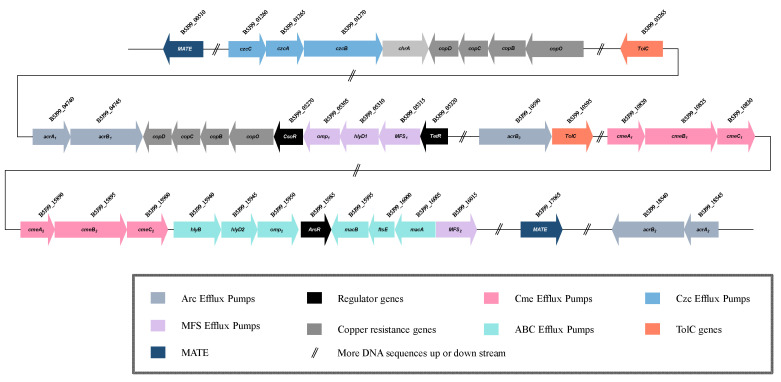
Genetic organization of RND, ABC and MFS type efflux pumps in the genome of strain *B. fulva* T2.

Bacterial efflux pumps are classified into seven distinct families or superfamilies: (I) ABC, the ATP-binding cassette superfamily; (II) RND, resistance-nodulation−cell-division superfamily; (III) MFS, the major facilitator superfamily; (IV) MATE, the multidrug and toxic compound extrusion family; (V) DMT, the drug/metabolite transporter superfamily; (VI) PACE, the proteobacterial antimicrobial compound efflux family; and (VII) AbgT, the *p*-aminobenzoyl-glutamate transporter family [43,44,45,46,47,48]. The quantity and type of efflux pumps vary significantly in bacterial lineages. A critical distinction in this regard is between Gram-positive and Gram-negative bacteria, which have fundamentally different cell envelope architectures and hence different requirements and capacities for small molecule export [49]. Members of the RND, ABC and MFS superfamilies have been shown to form tripartite complexes with periplasmic adapter proteins and outer-membrane proteins in Gram-negative bacteria, facilitating substrate efflux through the outer membrane [43,45,46,47,48]. Eight different efflux pumps have been discovered in the genome of strain T2 (Figure 7), including two CmeABC pumps, two AcrAB-TolC pumps and one CzcABC pump of the RND type; one MacAB-TolC pump and one HylBD-OMP pump of the ABC type; and one MFS-HylD-OMP pump of the MFS type.

#### 2.4.1. RND Type

##### CmeABC Efflux System

**Figure 8:**
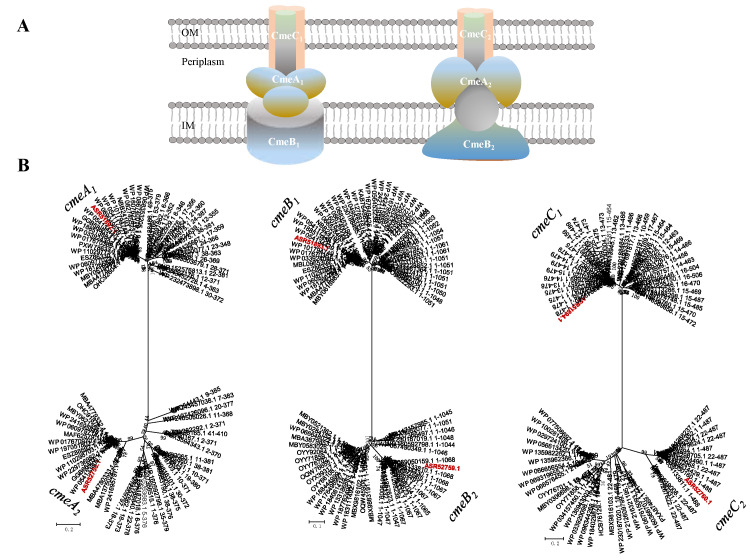
(**A**) Schematic representation of two tripartite CmeABC efflux pumps. (**B**) The minimum evolution phylogenetic trees of the *B. fulva* T2 CmeABC and related sequences. The phylogenetic tree for the CmeABC proteins was constructed using a minimum-evolution method. The tree was generated from multiple sequence alignments of protein sequences. The nodes represent bootstrap values based on 1000 replicates and the scale bar indicates 0.2 changes per position for *cmeA* and *cmeC*, and 0.1 for *cmeB*. Taxa accession numbers correspond to the NCBI database. The sequences derived from *B. fulva* T2 were indicated in the red.

Three-gene operon *cmeABC* is a tripartite efflux system containing the fusion protein *CmeA*, the inner membrane protein *CmeB* and the outer membrane protein *CmeC,* which confers resistance to a range of antibiotics, heavy metals, bile salts and other antimicrobial agents [50]. Two *cmeABC* gene clusters consisting of *cmeA_1_* (B5J99_10820), *cmeA_2_* (B5J99_15890), *cmeB_1_* (B5J99_10825), *cmeB_2_* (B5J99_15895), *cmeC_1_* (B5J99_10830) and *cmeC_2_* (B5J99_15900) genes were found in the genome of *B. fulva* T2 (Figure 7). Genomic analysis of strain *B*. *fulva* T2 revealed the presence of two putative *cmeABC* efflux pump operons closely homologous to that of *Sphingomonas wittichii* RW1 [51]. This locus contains the *cmeA_1_* gene putatively encoding the fusion protein functioning as the periplasmic adaptor protein CmeA showed 59.3% of similarity (48.7% for *cmeA_2_*) to that of *S. wittichii* RW1, the *cmeB_1_* gene encoding the inner-membrane RND protein CmeB showed 62.5% of similarity (58.1% for *cmeB_2_*) to *S. wittichii* RW1 followed by the *cmeC_1_* gene encoding outer-membrane CmeC which showed 50.4% of similarity (44.8% for *cmeC_2_*) to that of *S. wittichii* RW1 (Table 3). Phylogenetic studies of CmeABC efflux proteins showed that *cmeABC* homolog genes formed clades with members of efflux fusion protein, inner membrane protein and outer membrane protein, respectively. Based on their placement in clades with *cmeABC* (Figure 8) and similarities with related proteins, it is likely that strain *B. fulva* T2 possesses two CmeABC pumps.

##### AcrAB-TolC Efflux System

**Figure 9:**
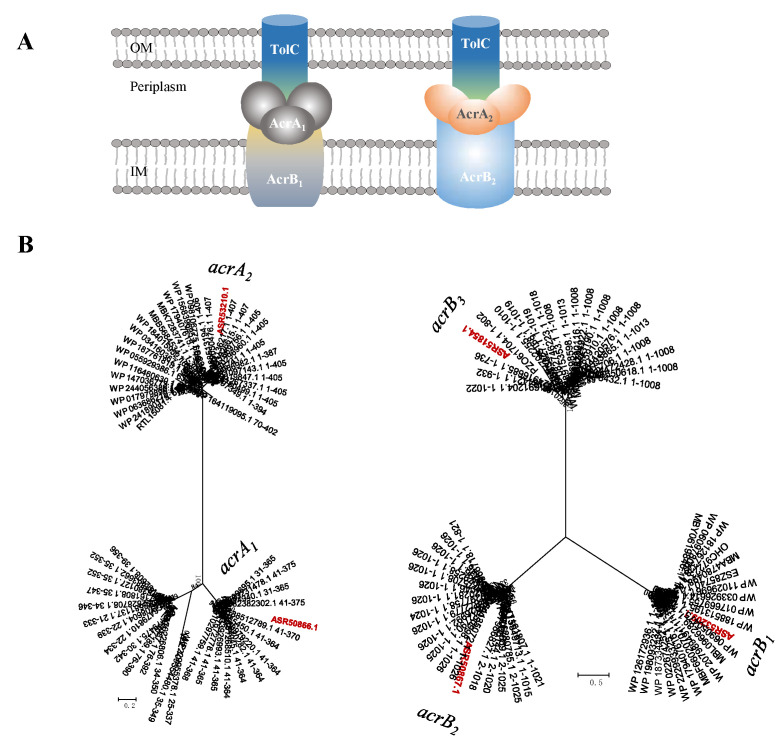
(**A**) Schematic representation of two tripartite AcrAB efflux pumps. (**B**) The minimum evolution phylogenetic trees of the *B. fulva* T2 AcrAB protein and related sequences. The nodes represent bootstrap values based on 1000 replicates and the scale bar indicates 0.2 changes per position for *acrA*, and 0.5 for *acrB*. Taxa accession numbers correspond to the NCBI database. The sequences derived from *B. fulva* T2 were indicated in red.

The AcrAB-TolC efflux system is known to be responsible for the extrusion of a wide variety of compounds in a number of Gram-negative bacteria, such as *E. coli*, *Salmonella*, *Klebsiella*, *Erwinia* and *Acinetobacter*. These compounds include antibiotics, lipophilic antimicrobial drugs, dyes, detergents and organic solvents [52]. The genome of strain T2 has been found to possess complete genes for two AcrAB-TolC efflux pumps, which are resistant to chloramphenicol, fluoroquinolone, tetracycline, novobiocin, rifampin, fusidic acid, nalidixic acid and *β*-lactam antibiotics [53]. Five genes *acrA_1_* (B5J99_04740), *acrA_2_* (B5J99_18545), *acrB_1_* (B5J99_04745), *acrB_2_* (B5J99_18540) and *acrA_3_* (B5J99_10590) encoding for inner-membrane and fusion proteins were observed, and these five genes along with the *tolC* gene which encodes outer-membrane protein make two AcrAB-TolC pumps (Figure 7). Genomic analysis revealed the presence of two putative *acrAB* efflux pump operons closely homologous to that of *Rhodospirillum centenum* SW, *Caulobacter crescentus* CB15 and *Erythrobacter litoralis* HTCC2594 [54,55,56]. The *acrA_1_* gene putatively encoding the acriflavin resistance protein A precursor working as the periplasmic adaptor protein AcrA had a 35.1% similarity to the membrane-fusion protein of *R. centenum* SW. Additionally, the *acrB1* gene encoding the acriflavin resistance protein B residing as the inner membrane RND protein AcrB displayed a similarity of 50.1% to that of *C.* *cres-centus* CB15. Another AcrAB pump was also identified, where the *acrA_2_* gene had 56.0% of similarity to that of *E. litoralis* HTCC2594, and *acrB_2_* had 72.9% of similarity to that of *E. litoralis* HTCC2594 (Table 3). Phylogenetic studies of AcrAB efflux proteins have revealed that *acrAB* homolog genes formed clades with members of efflux fusion proteins and inner membrane proteins, respectively. In this efflux system, TolC serves as an outer membrane protein which interacts with inner membrane efflux proteins to expel antibiotics or export virulence factors from bacteria [57]. The *tolC* gene also works in combination with other RND, ABC and MFS efflux pumps [58,59], and the genome of strain T2 possesses two *tolC* genes encoding outer-membrane proteins, presumably working cooperatively as outer-membrane proteins of AcrAB-TolC, MacAB-TolC and other two pumps. Based on their placement in clades with *acrAB* (Figure 9) and similarities with related proteins, strain *B. fulva* T2 is likely to possess two AcrAB-TolC pumps.

##### CzcABC Efflux System

**Figure 10:**
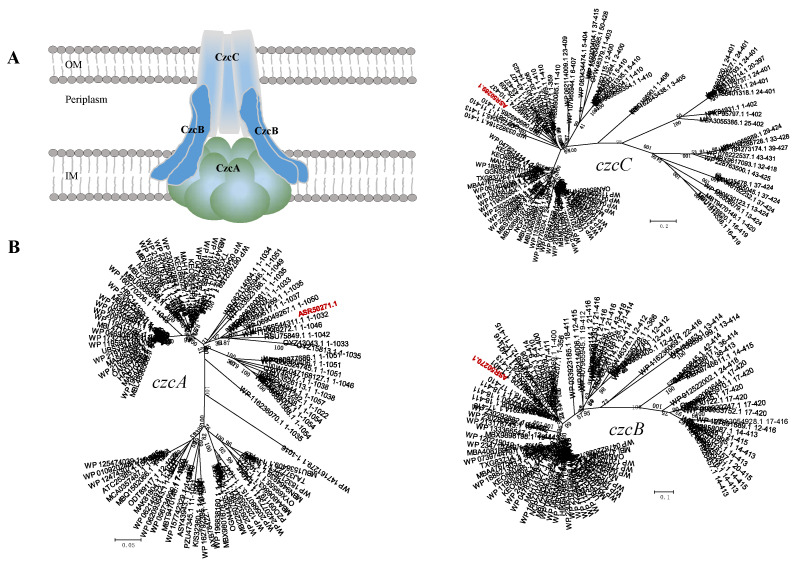
(**A**) Schematic representation of a tripartite CzcCBA efflux pump. (**B**) The minimum evolution phylogenetic trees of the *B. fulva* T2 CzcCBA protein and related sequences. The nodes represent bootstrap values based on 1000 replicates and the scale bar indicates 0.2 changes per position for *czcC*, 0.1 for *czcB* and 0.05 for *czcA*. Taxa accession numbers correspond to the NCBI database. The sequences derived from *B. fulva* T2 were indicated in red.

Microorganisms may be exposed to a variety of exogenous environmental toxins, such as hydrocarbons and heavy metals, which may be of natural or anthropogenic origin and may be harmful if allowed to accumulate in bacterial cells [60]. Efflux pumps play key roles in the removal of these substrates. CzcCBA belongs to the family of heavy metal efflux (HME) RND pumps and are involved mainly in response to the export of Co^2+^, Zn^2+^ and Cd^2+^ (czc) [61]. Three-gene operon *czcCBA* is also a tripartite efflux system consisting of the three proteins CzcC, CzcB and CzcA. The genome possesses the *czcC* gene (B5J99_01260) that encodes the heavy metal RND efflux outer-membrane protein CzcC, the *acrB* gene (B5J99_01265) encoding metal cation efflux fusion protein CzcB and the *czcA* (B5J99_01270) encoding for heavy metal RND efflux inner-membrane protein CzcA, which form a CzcCBA pump (Figure 7). Comparative genomic analysis revealed that this putative *czcCBA* efflux pump operon is closely homologous to that of *Caulobacter crescentus* CB15 [55], where the *czcC* gene had 37.0% of similarity to the cobalt–zinc–cadmium resistance gene of *C. crescentus* CB15, the *czcB* showed 56.4% of similarity to that of *C. crescentus* CB15 and the *czcA* gene showed 71.3% of similarity to that of *C. crescentus* CB15 (Table 3). Phylogenetic studies of CzcCBA efflux proteins have revealed that *czcCBA* homolog genes formed clades with members of efflux fusion proteins and inner-membrane proteins, respectively. Based on their placement in clades with *czcCBA* (Figure 10) and similarities with related proteins, the strain *B. fulva* T2 is likely to possess a CzcCBA pump.

#### 2.4.2. ABC Type

ABC transporters are a family of membrane proteins that mediate different ATP-driven transport activities. Transporters classified within the ABC superfamily are ubiquitous to all domains of life and are likely to be the most abundant superfamily of transport proteins on Earth [62]. Transporters belonging to this family are known to be responsible for uptake or efflux of substrates like vitamins, amino acids, lipids, peptides, ions and drugs. Two ABC-type efflux pumps, namely MacAB-TolC and HlyBD-OMP, are present in the genome of *B. fulva* T2 (Figure 7). The MacAB-TolC pump is known to have resistance to a variety of macrolides, aminoglycosides and polymyxins [63].

##### MacAB-TolC Efflux System

**Figure 11:**
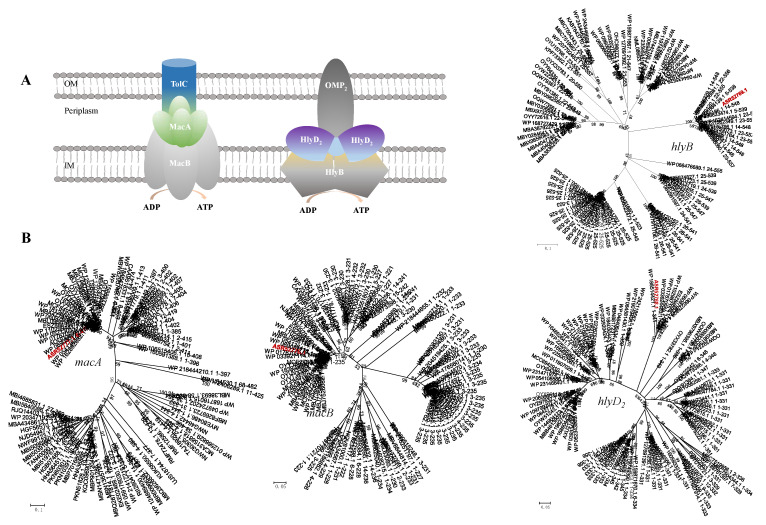
(**A**) Schematic representation of two tripartite ABC-type efflux pumps. (**B**) The phylogenetic trees were constructed using minimum-evolution method. The nodes represent bootstrap values based on 1000 replicates and the scale bar indicates 0.1 changes per position for *macA_1_* and *hlyB,* 0.05 for *macB* and *hlyD_2_*. Taxa accession numbers correspond to the NCBI database. The sequences derived from *B. fulva* T2 were indicated in red.

Here, the *macB* gene encodes for an ABC superfamily half-transporter, which combines with the MacA periplasmic fusion protein that binds to the outer-membrane channel TolC. The *macB* gene (B5J99_15995) encodes the macrolide export ATP-binding protein MacB, and the putative protein sequences exhibits 46.4% of amino acid sequence similarity to *Rhodospirillum centenum* SW [54]. The *macA* gene (B5J99_16005) encoding periplasmic adapter protein MacA shared 43.4% of sequence similarity with that of *Geobacter sulfurreducens* PCA [64]. A *ftsE* gene was also identified (Figure 7), encoding a cell-division signaling protein that is part of the cytoplasmic ATP-binding component MacB. This gene shared 61.6% of similarity with the *ftsE* gene of *Geobacter uraniireducens* Rf4 (CP000698). In this efflux system, TolC acts as an outer-membrane protein to form the efflux pump. The phylogenetic analysis for the MacBA homolog associated with the amino acid sequence of the macrolide export proteins was performed (Figure 11). The MacBA homolog forms a clade with related proteins and likely possesses a MacBA-TolC pump.

##### HlyBD_2_-OMP_2_ Efflux System

Another ABC-type tripartite pump HlyBD_2_-OMP_2_ was detected in the genome of *B. fulva* T2 (Figure 11). The gene *hlyB* (B5J99_15940) encoding putative inner-membrane protein HlyB, which acts in concert with the adaptor HlyD to export the large protein toxin, hemolysin from the cytoplasm across both membranes in a concerted step, had 51.8% of similarity to the toxin secretion ABC transporter protein of *Novosphingobium aromaticivorans* DSM 12444 (CP000248); *hlyD_2_* (B5J99_15945), which encodes the RND efflux membrane fusion protein, had 57.5% of similarity to that of *N. aromaticivorans* DSM 12444; *omp_2_* (B5J99_15950), which encodes the outer-membrane protein had 48.1% of similarity to that of *N. aromaticivorans* DSM 12444 (Table 3). Phylogenetic studies of HlyBD_2_-OMP_2_ efflux proteins have revealed that *hlyBD*-*omp_2_* homolog genes formed clades with members of efflux fusion proteins and inner membrane proteins, respectively. The phylogenetic analysis for the HlyBD homolog associated with the amino acid sequence revealed that the homologs form a clade with related proteins and likely possesses a HlyBD_2_-OMP_2_ pump.

#### 2.4.3. MFS Type: MFS-HlyD-OMP Efflux System

**Figure 12:**
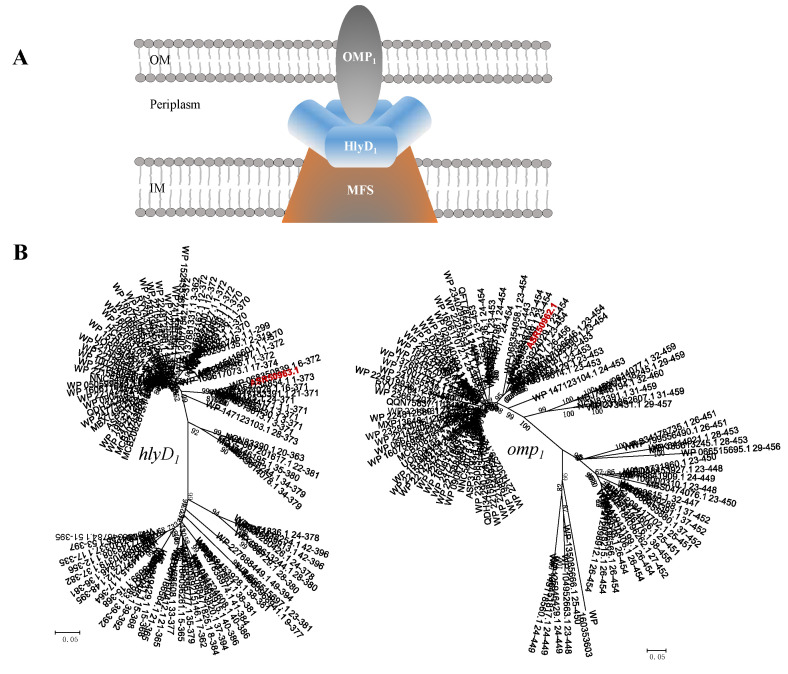
(**A**) Schematic representation of a tripartite MFS efflux pump. (**B**) The phylogenetic trees were constructed using minimum-evolution method. The nodes represent bootstrap values based on 1000 replicates and the scale bar indicates 0.05 changes per position for *hlyd_1_* and *omp_1_*. Taxa accession numbers correspond to the NCBI database. The sequences derived from *B. fulva* T2 were indicated in red.

MFS is also known to form tripartite complexes with periplasmic fusion proteins and outer-membrane proteins to facilitate substrate efflux across the outer membrane. A tripartite pump MFS_1_-HlyD_1_-OMP_1_ was also observed in the genome of *B. fulva* T2 (Figure 12). This gene cluster includes the *MFS_1_* gene (B5J99_05315) putatively encoding the inner membrane protein MFS transporter protein locating at inner membrane, *hlyD_1_* (B5J99_05310) that encodes HlyD family secretion protein as fusion protein and *omp_1_* that encodes the outer-membrane protein. A TetR family regulator gene *TetR* was present just upstream of the operon, downregulating the pump expression. The genomic analysis revealed the presence of the MFS_1_-HlyD_1_-OMP_1_ efflux pump operon closely homologous to that of *Sphingomonas wittichii* RW1 [51]. The comparative analysis showed that the gene *MFS_1_* had 39.5% of similarity to that of the *S. wittichii* RW1, *hlyD_1_* and *omp_1_* gene which had 54.9% and 53.2% of similarity to that of *S. wittichii* RW1, respectively (Table 3). Phylogenetic analysis of MFS efflux proteins revealed that all three genes formed clades with members of efflux fusion proteins and inner-membrane proteins, respectively. Based on their placement in clades with related proteins, the strain *B. fulva* T2 probably possesses an MFS-type pump.

Bacteria and other microorganisms have developed the ability to mediate the efflux of small molecule substrates and ions. Efflux pumps are able to transport diverse small molecules out of the cell. Transporters in Gram-negative bacteria typically have the broadest substrate recognition profiles, and it is conceivable that these pumps in particular could recognize and transport substrates such as amino acids and other metabolites. This analysis reveals that the genome of *B. fulva* T2 encodes a variety of transport proteins required for the export of toxic compounds and the import of essential molecules such as sugars, amino acids, ions and peptides. The classification of transporters into categories such as RND, ABC or MFS transporters does not necessarily indicate anything about their function in vivo. However, molecular genetics is useful for gaining initial insights into the significance of transporters to bacterial physiology. In addition, a comprehensive biochemical analysis is required to completely understand the biological roles of RND-, ABC- and MFS-type transporters and other transport systems, as well as to determine their action mechanisms.

## 3. Materials and Methods

### 3.1. Isolation and Culture Conditions

*Blastomonas fulva* T2 was isolated from a *Microcystis* culture in Daejeon, Republic of Korea, using dilutions to extinction (10^6^ or 10^7^) method in R2A medium (Difco, Franklin Lakes, NJ, USA) at 25 ^◦^C for 7 days. *Microcystis* cells were grown in 500 mL standard cell culture flasks using Blue–Green (BG11) broth (Merck, St. Louis, MO, USA) under the following conditions: 20 °C, 35% humidity, 12 h:12 h light-dark photoperiod, 20 μmol m^−2^ s^−1^ irradiance and 200 rpm agitation. A 100 L subsample of the suspended material from the *Microcystis* culture was aseptically disseminated onto R2A agar under heterotrophic conditions. The isolated strain T2 then was routinely sub-cultivated on R2A agar at 30 °C for 48 h and kept in a glycerol solution (20%, *v*/*v*) at −70 °C for long-term preservation.

### 3.2. Phylogenetic and Genomic Analyses

Genomic DNA was extracted using the FastDNA^TM^ SPIN DNA-extraction kit according to the manufacturer’s instructions and purity was checked using a ND2000 spectrometer (Nanodrop Technologies, Inc., Wilmington, DE, USA). PCR amplification of the *pufLM* genes was performed following the method previously described [32,34]. Genomic sequencing was performed using the PacBio RS II (Pacific Biosciences, Menlo Park, CA, USA) and the Illumina HiSeq platform at Macrogen (Seoul, Korea). SMRT Link v5.0.1 was used to do the sequence quality control of reads filtration and the assemblage [65]. The genome was annotated and compared in the RAST pipeline and the SEED Viewer, respectively [66,67], where protein functions were defined in the FIGfam collection [68]. The predicted protein coding sequences (CDSs) were compared to the COGs (Clusters of Orthologous Groups) database (http://www.ncbi.nlm.nih.gov/COG/, May 2022) to determine the functional category and summary statistics [69,70]. Phylogenetic analysis of protein sequences was performed using MEGA 7.0 [71]. Amino acid sequence alignment was done using the programs CLUSTAL X (version 1.8) [72], and the phylogenetic trees were reconstructed using the algorithm of minimum-evolution (ME) [73]. Bootstrap values of the phylogenetic trees were calculated on 1000 resamplings of the sequences [74]. For trees of *mlr*-related and multidrug efflux system proteins, the amino acid sequences were used as queries in blastp searches to identify other homologs. 

### 3.3. Microcystin Assay

For MC degrading assays, strain T2 was cultivated in 500 mL culture flasks in R2A medium containing 20 μg·L^−1^ MC-LR (Supelco, Belfort, PA, USA) with constant shaking at 150 rpm at 30 °C for 24 h [75]. The cell growth was measured at OD 600 nm and cell culture was harvested at 12 h and 24 h. The supernatant was filtered through a 0.22 μm polycarbonate filter after 10 min of centrifugation at 10,000× g. The MC was quantified using QuantiPlate™ Kit (Envirologix, Inc., Portland, ME, USA). All experiments were performed in triplicate.

## 4. Conclusions

The genome of *B. fulva* T2 represents the first detailed analysis of a genome from a species of *Blastomonas* that grows optimally at moderate temperatures and neutral pH. To summarize our investigations, the strain T2 from a *Microcystis* culture belonging to the genus *Blastomonas* was studied through genomic analysis, which confirmed its capability to degrade MC-LR. A 7.9K gene cluster containing *mlrA*, *mlrB*, *mlrC* and *mlrD_1,2,3,4_* genes is involved in the degradation of microcystin. Like other members in the genus *Blastomonas*, phototrophic systems were detected. *B. fulva* T2 also contains genes encoding for proteins of RND-, ABC- and MFS-type multidrug efflux systems. Several efflux pumps have been identified in a single bacterial cell, and multiple efflux pumps may have additive or greater-than-additive effects on drug resistance and substrate transport. The analysis of genes and their function is challenging, because it is frequently impeded by protein preparations necessary for in vitro experiments or the determination of the three-dimensional structure of transporters. Pathogenic and nonpathogenic bacterial genomes contain multiple uncharacterized transporters that may be essential for the growth and/or survival of these organisms and should therefore not be disregarded. The genome sequence and comparative genome analyses of *B. fulva* T2 provide a genetic blueprint and physiological characteristics which help us to understand the different metabolism and evolutionary features of the genus *Blastomonas*, especially the multidrug efflux system in non-pathogenic bacteria.

## Figures and Tables

**Table 1:** General features of strain *B. fulva* T2.

Item	Description
General feature	
Classification	Domain *Bacteria*
	Phylum *Proteobacteria*
	Class *Alphaproteobacteria*
	Order *Sphingomonadales*
	Family *Sphingomonadaceae*
	Genus *Blastomonas*
Type strain	KCTC 42354^T^
Gram stain	Negative
Morphology	Rods
Motility	Non-motile
Temperature range	10–37 °C
Salinity range	NaCl, 0 to 1%
pH range	6–8
Project name	PRJNA377807
Geographic location	South Korea
Collection date	June-15, 2014
Environment (biome)	Freshwater microalgae
Isolation source	Co-culture of microalgae
Sequencing	
Sequencing platform	PacBio RS II with P6-C4 chemistry
Assembler SMRT Analysis v2.3.0	SMRT Analysis v2.3.0
Annotation source	Prokka v1.13

**Table 2:** Similarities between *mlr*-like genes of *B. fulva* T2 and other available *mlr* sequences in GenBank. NT, nucleotide; AA, amino acid.

Genes	Locus Tag (Length, bp)	Description	Similarity (NT, %)	Similarity (AA, %)	Related Taxa
*mlrA*	B5J99_03485 (945)	Microcystin degrading enzyme, MlrA	51.9	40.0	*Novosphingobium* sp. THN1
		Microcystin degrading enzyme, MlrA	51.8	39.3	*Sphingomonas* sp. ACM-3962
		Microcystin degrading enzyme, MlrA	95.4	38.5	*Stenotrophomonas* sp. EMS
		Microcystin degrading enzyme, MlrA	49.1	40.3	*Novosphingobium* sp. MD-1
		Microcystin degrading enzyme, MlrA	51.5	42.2	*Sphingosinicella* sp. JEZ-8L
*mlrB*	B5J99_03460 (1644)	Microcystin degrading enzyme, MlrB	49.5	27.5	*Sphingopyxis* sp. X20
		Microcystin degrading enzyme, MlrB	49.2	27.1	*Sphingomonas* sp. ACM-3962
		Microcystin degrading enzyme, MlrB	49.4	27.7	*Sphingopyxis* sp. MB-E
		Microcystin degrading enzyme, MlrB	49.9	27.3	*Sphingopyxis* sp. C-1
		Microcystin degrading enzyme, MlrB	49.0	31.3	*Novosphingobium* sp. MD-1
*mlrC*	B5J99_03490 (834)	M55 family metallopeptidase	61.0	53.7	*Steroidobacter cummioxidans* 35Y
		D-aminopeptidase, DppA	55.8	54.0	*Sphingosinicella microcystinivorans* B9
		D-aminopeptidase, DppA	59.5	52.9	*Sphingomonas* sp. Y57
		D-aminopeptidase, DppA	55.8	54.0	*Sphingosinicella microcystinivorans* DSM 19791
		M55 family metallopeptidase	58.2	42.1	*Paucibacter toxinivorans* DSM 16998
*mlrD_1_*	B5J99_03465 (774)	ABC transporter ATP-binding protein	99.4	99.2	*Blastomonas* sp. AAP25
		ABC transporter ATP-binding protein	85.8	89.1	*Erythrobacter ramosus* DSM 8510
		Dipeptide transporter ATP-binding subunit	63.7	57.4	*Citreicella* sp. C3M06
		ABC transporter ATP-binding protein	62.2	56.5	*Bosea* sp. 32-68-6
		Dipeptide transporter ATP-binding subunit	60.3	56.3	*Inquilinus limosus* Inq sc_033
*mlrD_2_*	B5J99_03470 (918)	ABC-type glutathione transport system ATPase	99.5	98.9	*Blastomonas* sp. AAP25
		ABC transporter ATP-binding protein	83.6	86.2	*Erythrobacter ramosus* DSM 8510
		ABC transporter ATP-binding protein	48.6	46.4	*Virgibacillus litoralis* DSM 21085
		ABC transporter ATP-binding protein	48.7	49.2	*Marinomonas pollencensis* CECT 7375
		ABC transporter ATP-binding protein	57.7	52.7	*Aliidongia dinghuensis* CGMCC 1.15725
*mlrD_3_*	B5J99_03475 (816)	ABC transporter permease	86.5	95.9	*Erythrobacter ramosus* DSM 8510
		ABC transporter permease	59.4	54.0	*Paucibacter toxinivorans* DSM 16998
		D, D-dipeptide ABC transporter permease	58.1	53.1	*Ensifer* sp. ZNC0028
		D, D-dipeptide ABC transporter permease	57.6	53.1	*Ensifer adhaerens* ST2
		D, D-dipeptide ABC transporter permease	57.4	52.7	*Mesorhizobium* sp. INR15
*mlrD_4_*	B5J99_03480 (1002)	ABC transporter permease	99.2	98.7	*Blastomonas* sp. AAP25
		ABC transporter permease	86.0	90.7	*Erythrobacter ramosus* DSM 8510
		ABC transporter permease	60.9	53.8	*Sphingomonadaceae* bacterium BROCD036
		ABC transporter permease	58.6	52.1	*Hypericibacter terrae* R5913
		D,D-dipeptide transport system permease, DdpB	56.2	49.7	*Advenella mimigardefordensis* DSM 17166

**Table 3:** Similarities between genes associated with microbial efflux systems of *B. fulva* T2 and other available genes in GenBank.

Genes	Locus Tag (Length, bp)	Description	Similarity (AA, %)	E-Value	Related Taxa
*cmeA_1_*	B5J99_10820 (1155)	Membrane fusion protein of RND family efflux pump	59.3	3 × 10^−12^	*Sphingomonas wittichii* RW1
*cmeA_2_*	B5J99_15890 (1197)	Membrane fusion protein of RND family efflux pump	48.7	3 × 10^−91^	*Sphingomonas wittichii* RW1
*cmeB_1_*	B5J99_10825 (3192)	Multidrug efflux RND transporter permease	62.5	0.0	*Sphingomonas wittichii* RW1
*cmeB_2_*	B5J99_15895 (3207)	Multidrug efflux RND transporter permease	58.1	0.0	*Sphingomonas wittichii* RW1
*cmeC_1_*	B5J99_10830 (1437)	Efflux transporter outer membrane protein	50.4	3 × 10^−12^	*Sphingomonas wittichii* RW1
*cmeC_2_*	B5J99_15900 (1467)	Efflux transporter outer membrane protein	44.8	2 × 10^−92^	*Sphingomonas wittichii* RW1
*acrA_1_*	B5J99_04740 (1150)	Membrane-fusion protein	35.1	3 × 10^−49^	*Rhodospirillum centenum* SW
*acrA_2_*	B5J99_18545 (1224)	Efflux system membrane fusion protein	56.0	3 × 10^−12^	*Erythrobacter litoralis* HTCC2594
*acrB_1_*	B5J99_04745 (3081)	RND multidrug efflux transporter	50.1	0.0	*Caulobacter crescentus* CB15
*acrB_2_*	B5J99_18540 (3183)	RND multidrug efflux transporter	72.9	0.0	*Erythrobacter litoralis* HTCC2594
*czcA*	B5J99_01270 (3153)	Cobalt/zinc/cadmium resistance protein	71.3	0.0	*Caulobacter crescentus* CB15
*czcB*	B5J99_01265 (1212)	Cobalt/zinc/cadmium efflux RND transporter,	56.4	3 × 10^−13^	*Caulobacter crescentus* CB15
*czcC*	B5J99_01260 (1245)	Heavy metal RND efflux outer membrane protein	37.0	6 × 10^−52^	*Caulobacter crescentus* CB15
*macA*	B5J99_16005 (1275)	Macrolide-specific efflux protein	43.4	6 × 10^−78^	*Geobacter sulfurreducens* PCA
*macB*	B5J99_15995 (1251)	Macrolide export ATP-binding/permease protein	46.4	2 × 10^−86^	*Rhodospirillum centenum* SW
*ftsE*	B5J99_16000 (713)	Macrolide export ATP-binding/permease protein	61.6	1 × 10^−69^	*Geobacter uraniireducens* Rf4
*hlyB*	B5J99_15940 (1659)	HlyB family ABC transporter	51.8	3 × 10^−15^	*Novosphingobium aromaticivorans* DSM 12444
*hlyD_2_*	B5J99_15945 (1053)	HlyD family efflux transporter periplasmic adaptor	57.5	2 × 10^−11^	*Novosphingobium aromaticivorans* DSM 12444
*omp_2_*	B5J99_15950 (1503)	Outer membrane protein	48.1	1 × 10^−11^	*Novosphingobium aromaticivorans* DSM 12444
*MFS_1_*	B5J99_05315 (1667)	MFS transporter	39.5	1 × 10^−11^	*Sphingomonas wittichii* RW1
*hylD_1_*	B5J99_05310 (1054)	HlyD family secretion protein	54.9	1 × 10^−11^	*Sphingomonas wittichii* RW1
*omp_1_*	B5J99_05305 (1287)	RND efflux system outer membrane protein	53.2	1 × 10^−12^	*Sphingomonas wittichii* RW1

## Data Availability

The complete genome sequence of *Blastomonas fulva* T2 has been deposited at GenBank under the accession number CP020083 (CP020084 for plasmid). The strain is available from two different culture collections, namely JCM (Japan Collection of Microorganisms) and KCTC (Korean Collection for Type Cultures), with the accession numbers KCTC 42354^T^ and JCM 30467^T^, respectively.
